# Predicting the cumulative medical load of COVID-19 outbreaks after the peak in daily fatalities

**DOI:** 10.1371/journal.pone.0247272

**Published:** 2021-04-01

**Authors:** Claudius Gros, Roser Valenti, Lukas Schneider, Benedikt Gutsche, Dimitrije Marković

**Affiliations:** 1 Goethe University Frankfurt, Frankfurt a.M., Germany; 2 Technische Universität Dresden, Dresden, Germany; The University of Adelaide, AUSTRALIA

## Abstract

The distinct ways the COVID-19 pandemic has been unfolding in different countries and regions suggest that local societal and governmental structures play an important role not only for the baseline infection rate, but also for short and long-term reactions to the outbreak. We propose to investigate the question of how societies as a whole, and governments in particular, modulate the dynamics of a novel epidemic using a generalization of the SIR model, the reactive SIR (short-term and long-term reaction) model. We posit that containment measures are equivalent to a feedback between the status of the outbreak and the reproduction factor. Short-term reaction to an outbreak corresponds in this framework to the reaction of governments and individuals to daily cases and fatalities. The reaction to the cumulative number of cases or deaths, and not to daily numbers, is captured in contrast by long-term reaction. We present the exact phase space solution of the controlled SIR model and use it to quantify containment policies for a large number of countries in terms of short and long-term control parameters. We find increased contributions of long-term control for countries and regions in which the outbreak was suppressed substantially together with a strong correlation between the strength of societal and governmental policies and the time needed to contain COVID-19 outbreaks. Furthermore, for numerous countries and regions we identified a predictive relation between the number of fatalities within a fixed period before and after the peak of daily fatality counts, which allows to gauge the cumulative medical load of COVID-19 outbreaks that should be expected after the peak. These results suggest that the proposed model is applicable not only for understanding the outbreak dynamics, but also for predicting future cases and fatalities once the effectiveness of outbreak suppression policies is established with sufficient certainty. Finally, we provide a web app (https://itp.uni-frankfurt.de/covid-19/) with tools for visualising the phase space representation of real-world COVID-19 data and for exporting the preprocessed data for further analysis.

## 1 Introduction

Epidemic outbreaks differ widely with respect to the parameters defining their dynamics and the societal impact, such as infection rate, fatality rate, and the rate of critical cases requiring hospitalization. In order to gauge the effectiveness and suitability of containment policies, one needs therefore to discern the parameters characterizing a given epidemic process. The precise assessment of these quantities is however difficult for novel pathogens, with the consequence that it is in practice a notoriously difficult task to predict disease spreading [1]. Outbreak case data is in addition often both noisy and biased at early stages [2], which implies that core epidemiological parameters cannot be estimated with sufficient precision. This is a hard limitation for epidemic forecasting, as small differences in dynamical parameters can lead to drastically different outcomes [3]. This problem also affects machine learning approaches to the COVID-19 pandemic [4, 5], albeit to a lesser extend.

However, in spite of the above limitations, it is safe to assume that both individuals and governments will react to the spread of a new infectious disease. Given the severity of the COVID-19 pandemic [6], it is not surprising that the rising case and fatality numbers not only forced governments to impose lock-down measures [7, 8], but also motivated people to avoid travelling and mass gatherings [9]. Hence, to understand the dynamics of COVID-19 outbreaks, we propose to model the feedback of spontaneous societal and imposed governmental restrictions using a standard epidemic model that is modified in one key point: the reproduction rate of the virus is not constant, but evolves over time alongside with the disease in a way that leads to a ‘flattening of the curve’ [10]. The basis of the proposed model is the SIR (Susceptible, Infected, Recovered) model, which describes the evolution of a contagious disease for which immunity persists substantially longer than the outbreak itself [11]. We extend the model by introducing a negative feedback loop between the severity of the outbreak and the initial reproduction rate *g*_0_. Our model contains two parameters, *α*_*X*_ and *α*_*I*_, which quantify respectively the amount of long- and short-term epidemic control. The first, *α*_*X*_, represents the contribution of the cumulative case count *X* to the negative feedback loop. For the second parameter, *α*_*I*_, short-term control, the growth rate is reduced when the current number of active cases, *I*, is large. The resulting process is denoted the controlled SIR model.

The controlled SIR model draws its motivation from previous epidemiology modeling. One of the first pieces of evidence showing that human behaviour affects spreading dynamics [12, 13], came from the study of measles epidemics [14]. Generalizations of the SIR model account for various effects of societal response to an outbreak, such as self-isolation [15], contact-frequency reduction and quarantine [16], changes in human mobility [17], together with the effects of geographic and societal networks [18], and of the explicit influence of voluntary social distancing on the epidemic [19]. For a detailed analysis, epidemiology models can be extended to cover a range of additional aspects [20], with an example being the distinction between symptomatic and asymptomatic cases [21]. These kind of complex models are in general not accessible to an explicit analytic handling. It has also been questioned, whether detailed modeling leads to improved predictions [1, 2], given that field data is inherently noisy. To this regard we will discuss the under-counting problem, namely that not all infections are detected, and its relation to the statistics of the deceased.

The controlled SIR model was recently introduced in [22], where the authors analysed long- and short-term contributions to the negative feedback loop separately, but not as arbitrary mixtures. In contrast, we derive here an analytic solution for the controlled SIR model in the presence of both long-term and short-term control. Additionally, we show that the description of COVID-19 field data is substantially improved when both short-term and long-term control are included. For data analysis and model validation, we use publicly available COVID-19 case and fatality counts for a wide range of countries and regions.

We find that the cumulative number of fatalities within a given period of a few weeks before the peak number of daily fatalities increases by 30% for the same period after the peak. Strikingly, this result is found to hold universally across all considered countries and regions. In contrast to the universality with regard to the increase in fatalities, substantial differences in the country-specific intrinsic reproduction factors and short- and long-term control parameters are found. A comprehensive theoretical description based on an analytic solution of the controlled SIR model is given, together with a detailed validation (based on simulated data) of the statistical inference used for estimation of country specific parameters. We also evaluate search-engine based measures quantifying the effectiveness of lock-down measures and the impact of structural factors (e.g. population density) on the infection rate and doubling time. Finally, we conclude that the controlled SIR model allows precise quantification of the outbreak dynamics, and provides a predictive framework for assessing the effectiveness of containment measures and future medical load.

## 2 Results

We let *X* denote cumulative case counts, both for field data and for theory results. For the number of new cases, which are typically reported in official COVID-19 datasets on a daily basis, the symbol Δ*X* is used. We will add a time tag subscript *d* to denote reported counts on a specific day. In that case, the following sum rule holds: $X_{d}=\sum_{\tilde{d}\leq d}\Delta X_{\tilde{d}}$. In analogy, we denote with *F* and Δ*F* respectively cumulative and daily fatalities. Importantly, the model presented here is explicitly defined for one isolated epidemic outbreak. Our analysis focuses thus exclusively on the initial outbreak of the COVID-19 pandemic, the first wave, as defined in more details in Sect. Data smoothing / peak definition.

### 2.1 Controlled SIR model

The logic of an infectious disease is described by the SIR model,

$$\begin{array}{c} \tau \dot{S}=-g\frac{S}{N}I,\;\;\tau \dot{I}=\left(g\frac{S}{N}-1\right)I,\;\;\tau \dot{R}=I\; \end{array}$$ (1)

which takes the number of susceptible *S* = *S*(*t*), infected *I* = *I*(*t*) and recovered (removed) individuals *R* = *R*(*t*), as dynamical variables [23]. The sum, *S* + *I* + *R* = *N*, is assumed to be constant at all times *t*, as the population size *N* remains approximately unchanged over the course of the outbreak. In its basic formulation, the SIR model is characterized by a timescale, *τ*, and a dimensionless reproduction factor, *g*.

Note that the Eq (1) describes an uncontrolled isolated outbreak in an environment that does not react to the disease. In reality, counter measures will be taken either spontaneously by the general public, or will be imposed by governmental institutions. As a result, the reproduction factor will fall below its intrinsic value, which we denote with *g*_0_. We make the assumption that reactions to the unfolding of the epidemic are based either on the current situation (the current active cases, *I*) or on the overall history of the outbreak (the total cases *X* = *N* − *S*). Formally, we can express this dependence as

$$\begin{array}{c} g=\frac{g_{0}}{1+\alpha_{X}\frac{X}{N}+\alpha_{I}\frac{I}{N}},\;\;\{\begin{array}{ccc} \alpha_{I}\geq 0 & : & \text{short-term}\;\text{control}\\ \alpha_{X}\geq 0 & : & \text{long-term}\;\text{control} \end{array} \end{array}$$ (2)

Note that the functional form in Eq (2) parallels the law of diminishing return [24], which reflects the intuition that containment becomes progressively harder.

**Fig 1:**
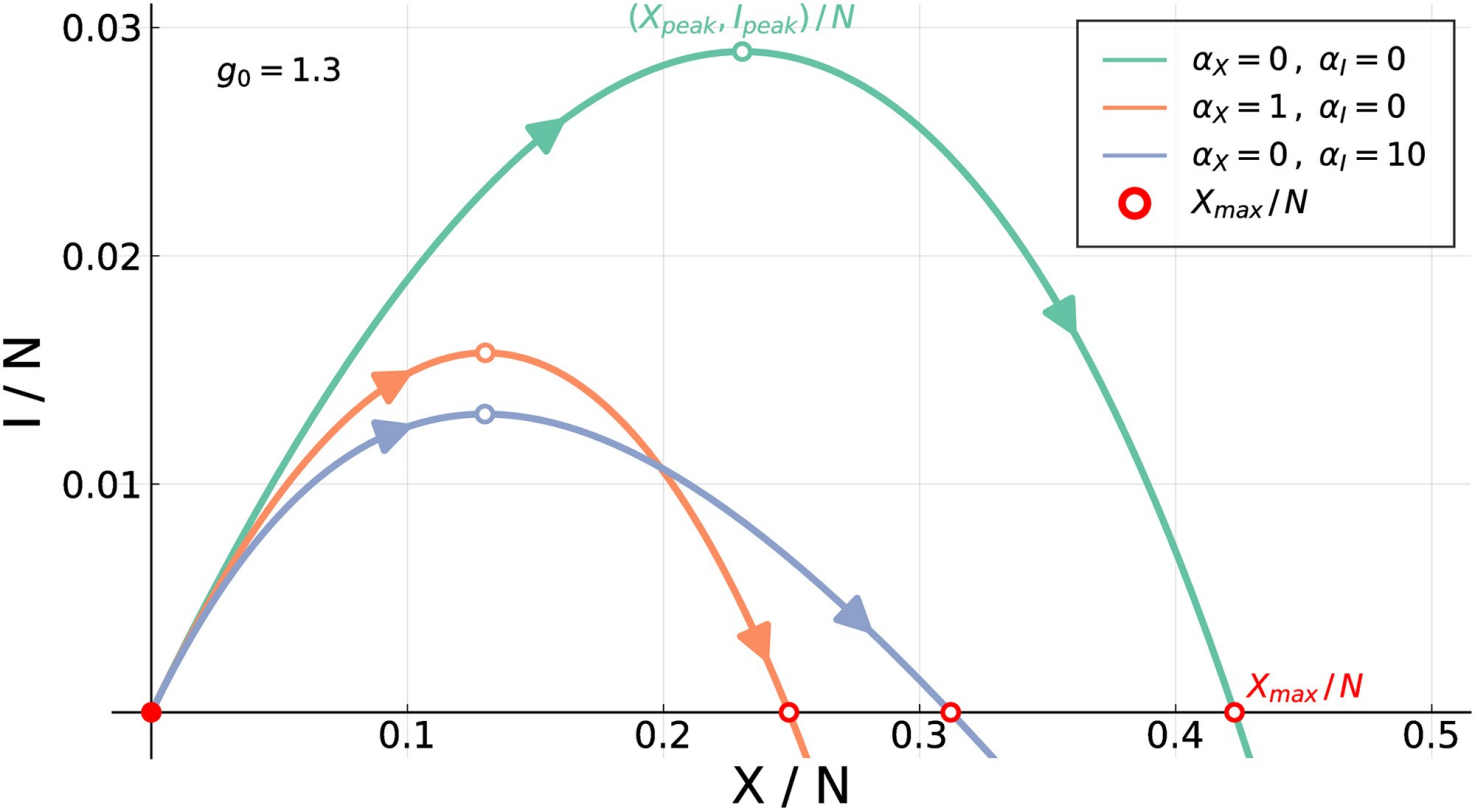
Short and long-term control. Outbreaks are contained when either short-term or long-term control is present, see Eqs (1) and (2). Long-term control (*α*_*X*_ > 0, *α*_*I*_ = 0) produces more symmetrically confined outbreaks than short-term control (*α*_*X*_ = 0, *α*_*I*_ > 0).

The controlled SIR model, Eq (1) together with Eq (2), can be solved analytically in phase space (see Sect. Exact solution of the controlled SIR model for more details). One obtains the following rigorous relation:

$$I(X)=\frac{g_{0}+\alpha_{X}}{g_{0}-\alpha_{I}}X+N(\frac{g_{0}+\alpha_{X}}{g_{0}-\alpha_{I}}+\frac{1+\alpha_{X}}{\alpha_{I}})[{\left(1-\frac{X}{N}\right)}^{\alpha_{I}/g_{0}}-1],$$ (3)

which we will use throughout this paper to investigate the evolution of an epidemic outbreak, as function of total case numbers *X*, with time parametrizing implicitly the functional dependence of *I* = *I*(*t*) on *X* = *X*(*t*). For an illustration see Fig 1. Note that both long- and short-term control reduce the severity of an outbreak with respect to the uncontrolled scenario, *α*_*X*_ = *α*_*I*_ = 0, however with distinct shapes for the resulting phase space trajectories. The phase space (XI) representation tends to be stretched for short-term control and parabola-like for long-term control [22].

The maximum of *I*, the peak rate *I*_peak_, is obtained for

$$\begin{array}{c} \frac{X_{\text{peak}}}{N}=1\;-\;[(\frac{g_{0}+\alpha_{X}}{g_{0}-\alpha_{I}})/(\frac{\alpha_{I}}{g_{0}}\;(\frac{g_{0}+\alpha_{X}}{g_{0}-\alpha_{I}}+\frac{1+\alpha_{X}}{\alpha_{I}})){]}^{\frac{g_{0}}{\alpha_{I}-g_{0}}}\;. \end{array}$$ (4)

From Eq (4) one obtains *I*_p*eak*_ via Eq (3).

### 2.2 Data validation

**Fig 2:**
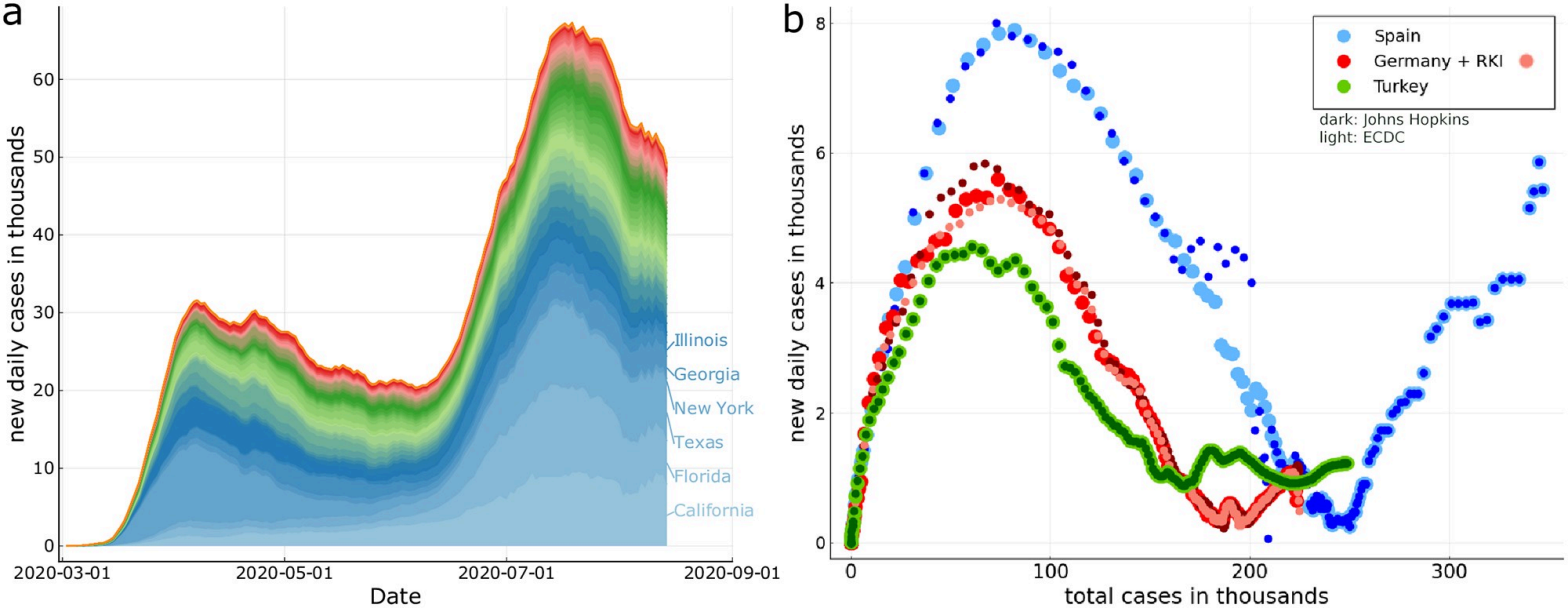
COVID-19 outbreak examples. *Left*: The timeline of the daily new infections for all US states, where the state with the biggest cumulative case count is set at the bottom, and the one with the lowest at the top. The curves are in part strongly asymmetric with respect to the time it takes for the outbreak to build up and to recede. A second, prominent peak is present. *Right*: Daily counts Δ*X* are plotted as a function of total counts *X*, which defines the XI-representation. For Spain, Germany and Turkey a comparison of ECDC (European Center of Disease Control), the Johns Hopkins, and the RKI (Robert Koch Institute, Germany) data. Seven-day moving averages have been used. Note the substantial scattering of the later-stage COVID-19 data for Spain, which is due to changes of official counting protocols. For the data sources see Data sources section.

As an example of the COVID-19 data examined we present in Fig 2 the timeline of the outbreak for all US states. Also shown is a comparison of several publicly available data sources (see Data sources section for details). For most daily values the Johns Hopkins and ECDC data agree, as illustrated in Fig 2b for the case of Spain, Turkey and Germany, which have been selected for illustrative purposes. For the latter, the case counts published by the German Robert Koch Institute have been added. Spain is a special case, as the official counting criteria did see a major revision end of May 2020 [25].

A key focus of the present study concerns the evolution of fatality counts. For the analysis we concentrated on countries, and states within the US, with cumulative death toll of at least 1000, a number which we found to allow for a robust analysis. The here proposed framework can be applied also to smaller outbreaks, albeit with the caveat of increased statistical fluctuations.

### 2.3 Fatalities rescaling

In practice, not all active cases (infected individuals) are detected and reported, with the consequence that the official numbers of daily cases, and likewise the total number, is subject to under-counting. Furthermore, even when an infected individual is identified, the report is normally delayed from the moment of the infection to the occurrence of symptoms, and subsequent positive testing, a process taking up to several weeks [26, 27]. Individuals identified as infected are most of the time isolated (quarantined) and the possibility that they further spread the disease is minimal. Hence, from the perspective of the outbreak dynamics, daily cases counts are an indicator for the number of individuals changing from the group of infectious to the removed individuals *R*, which are the ones unable to spread the disease.

Miscounting is present also for official fatality counts, but to a reduced extent [28]. It is possible to estimate the extent to which the history of fatalities and infections trace each other in phase space, by comparing the functional dependence of (*X*, Δ*X*) and (*F*, Δ*F*):

$$\begin{array}{c} \left(X,\Delta X\right)\;↔\;f_{F}\;\left(F,\Delta F\right)\;. \end{array}$$ (5)

A rationale for this procedure is presented within the Sect. Approximate integration.

**Fig 3:**
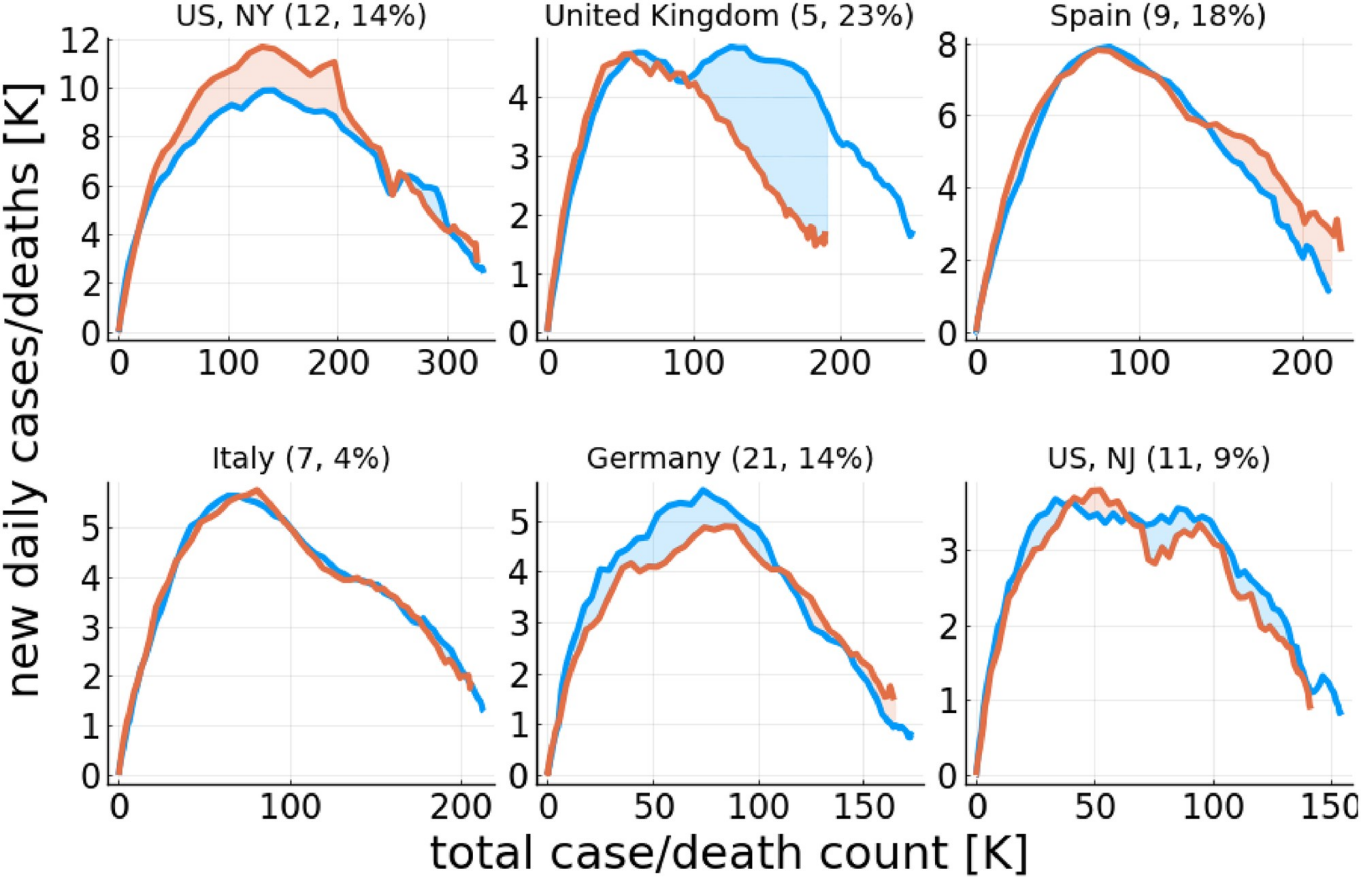
COVID-19 cases vs. fatalities. Daily new cases Δ*X* as a function of the total case count, *X* (blue), and the rescaled daily fatalities, Δ*F* → *f*_*F*_Δ*F*, as a function of the rescaled total death count, *F* → *f*_*F*_ *F* (orange). The rescaling factors *f*_*F*_, given in brackets (first number), have been determined by aligning the initial slopes Δ*F*/*F* and Δ*X*/*X*. The accuracy of the rescaling, *S*_*F*_, measured as the relative area difference (shaded area over total area, see Eq (6)), is given in the brackets (second number). Shown are countries and regions with the highest cumulative fatality counts, out of the ones considered here, as listed in Table 1. The data has been terminated once Δ*X* has fallen by 70%, which we use to define the first outbreak.

In Fig 3 we show the relationship between daily cases and daily fatalities for the countries and US states with the highest death tolls. For some countries, like Italy, the rescaling procedure defined in Eq (5) works surprisingly well. The accuracy can be gauged by evaluating

$$\begin{array}{c} S_{F}=\frac{1}{X_{\text{tot}}}\sum_{d}\;|\Delta X_{d}-f_{F}\Delta F_{d}|\;, \end{array}$$ (6)

which corresponds to the percentage-wise miscounting of the daily cases Δ*X*_*d*_ with respect to rescaled daily fatalities *f*_*F*_Δ*F*_*d*_. Note that *S*_*F*_ → 1 when the rescaling factor *f*_*F*_ is set to zero, since *X*_tot_ = ∑_d_ Δ*X*_d_ and |Δ*X*_*d*_| = Δ*X*_*d*_.

In Table 1 the scaling accuracies in terms of *S*_*F*_ are listed for all countries and US state examined. Values of the order 10%-20% are typical. The quality of the matching suggests that the respective under-counting factors are stable, and not changing substantially over time. The results presented in Fig 3 indicate also that other factors, like the success of medical treatments, seem to have changed comparatively little over the observation period, here *d* < *d*′, where the *d*′ is the cut-off date for the first peak defined in Data smoothing / peak definition.

**Table 1:** Model and data analyses parameters. The effective reproduction factor *g*_0_ estimated from the (*F*, Δ*F*) representation, the relative fraction *L*/(*L* + *S*) of long-term control, defined by Eq (8), together with the rescaling factor *f*_*F*_ and the accuracy *S*_*F*_ of fatalities to case-count scaling, defined by Eq (6). The equivalent accuracies of the fits presented in Fig 4 are given by *S*_fit_. Also listed is the timescale *T*_*τ*_ used for evaluating *F*_before_/*F*_after_ (the number of fatalities per time before/after the peak) in Fig 5, and the cut-off date *d*′, given by the date at which the daily new cases Δ*X* of the first peak of the COVID-19 outbreak have dropped by 70% with respect to the maximum.

Region	*g*_0_	$\frac{L}{L+S}$	*f*_*F*_	*S*_*F*_	*S*_fit_	*T*_*τ*_	*d*′
Canada	1.17	0.34	11	0.12	0.09	29	2020-06-08
France	1.30	0.51	5	0.10	0.07	19	2020-04-29
Germany	1.21	0.49	21	0.14	0.06	20	2020-05-15
Italy	1.28	0.31	7	0.04	0.04	21	2020-05-06
Netherlands	1.31	0.29	7	0.16	0.06	17	2020-05-11
Portugal	1.26	0.29	23	0.18	0.15	20	2020-06-05
Romania	1.15	0.41	14	0.12	0.15	28	2020-06-05
Spain	1.39	0.32	9	0.18	0.03	16	2020-05-01
Turkey	1.19	0.52	35	0.07	0.07	24	2020-05-18
UK	1.28	0.34	5	0.23	0.03	20	2020-05-28
US, GA	1.27	0.09	25	0.21	0.14	30	2020-06-29
US, IL	1.12	0.37	21	0.18	0.09	36	2020-06-24
US, MA	1.22	0.33	13	0.15	0.08	25	2020-06-04
US, MI	1.24	0.39	10	0.17	0.08	22	2020-05-22
US, NJ	1.27	0.29	11	0.09	0.06	20	2020-05-22
US, NY	1.28	0.47	12	0.14	0.06	17	2020-05-07
US, PA	1.16	0.48	12	0.16	0.15	29	2020-06-06

The rescaling factors *f*_*F*_ reported in Table 1 have been determined separately for each country and US state by aligning the initial slopes Δ*X*/*X* and Δ*F*/*F*. The rationale is that the initial phase of an outbreak corresponds to the exponential-growth phase, for which the rescaling has to hold when circumstances do not change. The reason is that both case and death counts increase in the exponential phase with the same doubling time, with the delay of the fatalities contributing multiplicative to the rescaling factor *f*_*F*_.

### 2.4 Modeling fatality dynamics with an effective SIR model

The data presented in Fig 3 indicates, as discussed above, that death and case counts of reported COVID-19 data, approximately rescaled death counts. It is hence of interest to examine to which extent one can extract the characteristics of an outbreak directly from the fatality counts, which tend to be more reliable. For this purpose one could add a variable *F* to the SIR model and evaluate fatalities directly from first principles. Here we use the fact that *I* and *F* are necessarily related (only infected can die), modulo a time lag, which becomes however irrelevant in the XI phase space representation, as illustrated in Fig 1. For this purpose, we use the following mapping between reported daily and total fatalities and the variables *X* = 1 − *S* and *I* of the SIR model, Eq (1):

$$\begin{array}{c} \left(X,I\right)\;↔\;{\tilde{f}}_{F}\;\left(F,\Delta F\right)\;. \end{array}$$ (7)

**Fig 4:**
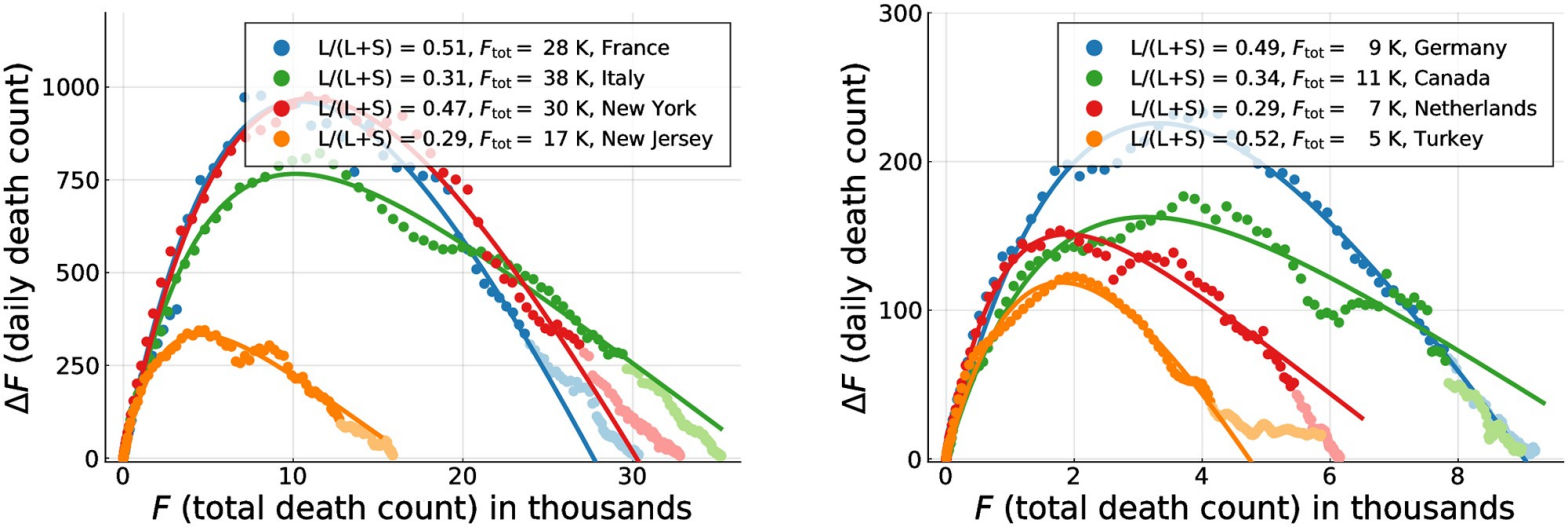
COVID-19 containment policies. Daily fatalities Δ*F* as a function of total death counts *F*. Comparison of data (seven-day centred averages, filled circles) and theory (lines). Points with lighter hue correspond to dates *d* > *d*′ not part of the parameter estimation. The theory corresponds to optimal fits of the exact solution (16) of the controlled SIR model, Eqs (1) and (2). The relative importance of long-term control, *L*/(*L* + *S*), as defined by Eq (8), is given, together with the model based estimate of the total death toll, *F*_tot_ (assuming a single COVID-19 peak/outbreak). Containment policy parameters for all countries and the degree of agreement between theory and data are presented in Table 1.

The rescaling factor defined here, ${\tilde{f}}_{F}$, is in general different from the one used in Eq (5). In Fig 4a direct comparison of the exact phase space trajectories (*X*, *I*) obtained for the controlled SIR model with reported death counts is presented. To this extent the development of Δ*F* vs. *F* in phase space has been fitted using the exact solution Eq (3) and an appropriated rescaling factor ${\tilde{f}}_{F}$ in Eq (7). Note that we have estimated the free model parameters only from the data associated to the initial outbreak, for dates *d* < *d*′, as explained in Data smoothing / peak definition. The data not considered for the loss function are indicated in Fig 4 by lighter hue markers.

Overall, the observed COVID-19 outbreaks can be described well using a mixture of long-term and short-term control, parametrized respectively by *α*_*X*_ and *α*_*I*_. An overview of the extracted parameters is presented in Table 1. It is important to recall that the growth factor *g*_0_ of the effective SIR model used for the description of the fatality dynamics in terms of an (*X*, *I*) representation does not correspond to the medical growth factor *R*_0_. Instead, the comparison presented in Fig 4 shows that it is possible to model the evolution of official fatality statistics directly in terms of an effective SIR model.

### 2.5 Tracing containment policies via fatality dynamics

The use of an effective SIR model to describe fatality statistics, as in Fig 4, allows to extract containment policies, the key rationale for this procedure. In absolute terms, the contributions *α*_*X*_ *X* and *α*_*I*_ *I*, to the reduction of *g*, vary strongly as functions of time. We use therefore the respective values at the peak of daily fatalities, which correspond via Eq (7) to the peak fraction *X*_peak_ of total cases, as given by Eq (4), and to the corresponding fraction of active cases, *I*_peak_.

Hence, we use the following relation

$$\begin{array}{c} \frac{L}{L+S}\equiv \frac{\alpha_{X}X}{\alpha_{X}X+\alpha_{I}I}{|}_{X=X_{\text{peak}},I=I_{\text{peak}}}\;, \end{array}$$ (8)

for a relative gauge, *L*/(*L* + *S*), that quantifies the fraction of control due to long-term control. Here *L* stands for ‘long’ and *S* for ‘short’. The extracted values of *L*/(*L* + *S*) are given in Table 1 together with the accuracy of the respective fits. For the countries shown in Fig 4 one observes, characteristically, that the epidemic decreases fast for countries with large fractions of long-term control, and slower when short-term control dominates. Long-term control is therefore substantially more efficient in containing an epidemic outbreak. This is also evident from the comparison given in S1 Fig between the two countries/regions with highest (Turkey) and lowest (USA/Georgia) fraction *L*/(*L* + *S*) of long-term control.

### 2.6 Universal fatality increase after the peak

People dying of a COVID-19 infection have been typically on intensive care beforehand, which implies that the medical load is roughly proportional to the number of fatalities incurring on a daily basis. Of interest is, in this regard, whether the average medical load decreases or increase after the peak of the outbreak has been reached, in particular when averaged over a timescale *T*_*τ*_ of several weeks.

**Fig 5:**
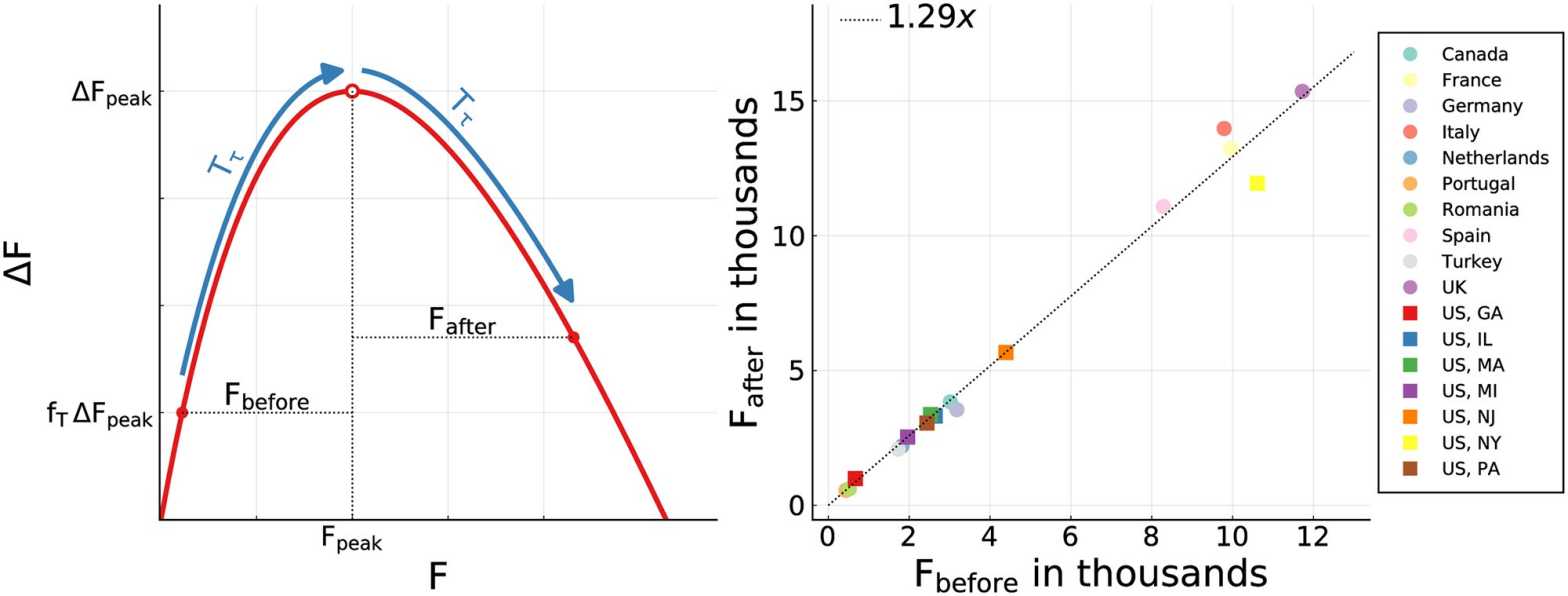
Fatalities before and after the peak—data. The observed numbers of fatalities *F*_before_ and *F*_after_ incurring over a time span *T*_*τ*_ before/after the peak of an epidemic outbreak. *Left*: Procedure illustration. See the Methods Section for the determination of *T*_*τ*_. *Right*: All observed ratios *F*_after_/*F*_before_ are close to 1.3. The linear fit corresponds to a linear regression with fixed intercept (*R*^2^ = 0.987). Per time, on average 30% more death incur after the peak.

We denote with *F*_before_, and respectively with *F*_after_, the number of deaths occurring in the *T*_*τ*_ days before/after daily fatalities peaked, $\Delta F_{\text{peak}}=I\left(X_{\text{peak}}\right)/{\tilde{f}}_{F}$, as determined by Eqs (3) and (7). The reference period *T*_*τ*_ is determined in our analysis by measuring the number of days that passed between *f*_*τ*_Δ*F*_peak_ and Δ*F*_peak_, that is between a small initial daily fatality count, *f*_*τ*_Δ*F*_peak_, and the peak medical load Δ*F*_peak_. See Fig 5 for an illustration. We took *f*_*τ*_ = 0.1 when possible, namely when the data for the same number of days after the peak was available and within the observation period. Otherwise the time span from Δ*F*_peak_ to the end of the reported timeline (or *d*′) was taken.

For all countries and US states examined, *F*_after_ is plotted as a function of *F*_before_ in Fig 5. One finds a near to perfect linear relationship

$$\begin{array}{c} F_{\text{after}}\approx 1.3\;F_{\text{before}}\;, \end{array}$$ (9)

which is quite remarkable. For the linear regression with fixed intercept we find *R*^2^ = 0.987. It implies, that the average medical load is predictably 30% higher after the peak, than before. Given that there is a time delay between the onset of an infection and the eventual fatality, a certain increase was to be expected. The finding that this holds for a wide range of countries and regions, is however highly non-trivial. This result facilitates in our view the planning for COVID-19 specific hospital capacities. The stable relationship between medical load before and after the peak fatalities is in particular surprising in the view that the functional developments of COVID-19 outbreaks vary considerably, as illustrated in Figs 3 and 4.

**Fig 6:**
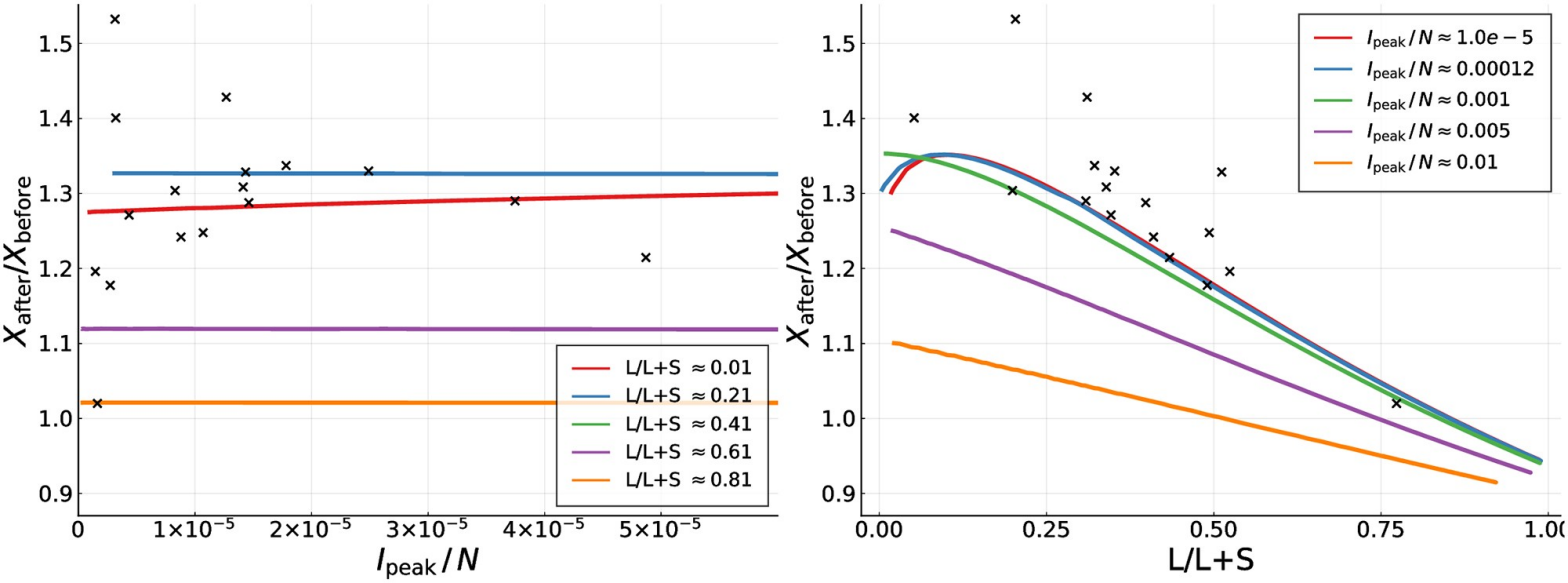
Fatalities before and after the peak—theory. For a fixed *g*_0_ = 1.25, this plot reveals simulation results (solid lines) and estimates from the data (crosses) for the ratio of average fatalities after and before the infection peak, *X*_after_/*X*_before_. Compare Fig 5. A large number of simulations of the controlled SIR model have been performed over a range of *α*_*X*_ and *α*_*I*_, which have been reordered subsequently in terms of the per population infection peak, *I*_peak_/*N*, and of the percentagewise contribution *L*/(*L* + *S*) of long-term control. For most countries *I*_peak_/*N* is typically of the order of 10^−4^ or smaller.

In Fig 6 we present the ratio *X*_after_/*X*_before_ of the cumulative numbers of cases occurring in the controlled SIR model during the above defined period *T*_*τ*_ before and after the peak. The theoretical estimates have been obtained keeping *g*_0_ = 1.25 fixed (see Eq (1)), scanning a wide range of *α*_*X*_ and *α*_*I*_. Note that the field data, which is also given, scatters somewhat around the 1.3 ratio, an effect which is not as evident when using alternative representations, as in Fig 5.

Given that case and fatality counts are related (re-scalable) for many countries, as illustrated in Fig 3, the results presented in Fig 6 can be understood as a first step towards an understanding why the ratio *F*_after_/*F*_before_ is of the order of 1.3 for the field data, as shown Fig 5. In fact one observes in Fig 6 that two conditions are necessary for *X*_after_/*X*_before_ to be of the order of 1.3, or slightly larger. Firstly, the per-population peak fraction of infected, *I*_*peak*_/*N* needs to be small, of the order of 10^−4^ or smaller, which is typically the case for field data. Secondly, control is dominated by short-term control, with long-term control contributing only in a minor way. This condition also holds, albeit only to a certain extent, given that *L*/(*L* + *S*) is generically smaller than 0.5; see Table 1 for details.

The data presented in Fig 6 indicates that the size of the relative infection count and the type of containment policy enacted influence relative medical loads. Further research is however necessary to clarify why *F*_after_/*F*_before_ ≈ 1.3 holds to the observed precision.

### 2.7 Influence of initial social distancing

Google compiled changes in search-engine queries that are indicative of increasing social distancing, with an example being a reduction of inquiries concerned with travelling to the workplace. Using an average of several indicators, we compiled the Google social distancing index (GSDI); see Google social distancing index (GSDI) for details. Numerically the index is gauged with respect to its pre-Corona value.

**Fig 7:**
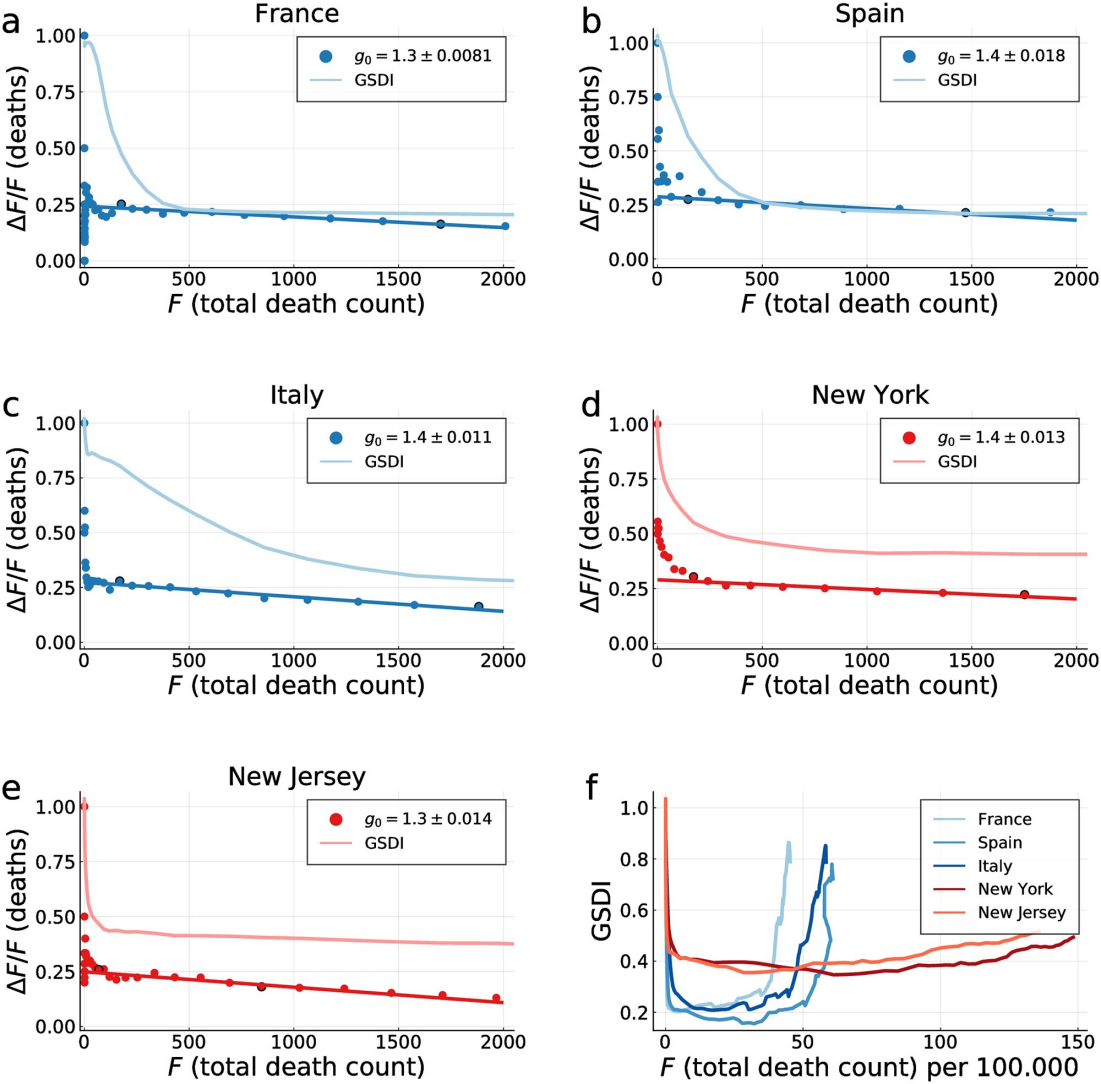
Fatalities vs. social distancing. Comparison of the Google social distancing index (GSDI), as defined in Google social distancing index (GSDI), with COVID-19 fatalities. **a)**-**e)** For France, Spain, Italy, New York and New Jersey. Shown is the ratio Δ*F*/*F* of the daily fatalities Δ*F* and the total death count *F* (filled circles), a linear fit between 2%–20% of the fatality peak (marked circles), and the respective GSDI. The slope has been used to calculate *g*_0_ in accordance to Eq (21). **f)** The GSDIs on an expanded scale, now as a function of fatalities per capita (per 100,000). The selected European countries and US states show distinct behaviors.

In Fig 7 we show the correlation between the GSDI and the ratio Δ*F*/*F* between reported daily fatalities Δ*F* and total fatalities *F*. As examples we selected countries and US states which head at least 10, 000 cumulative fatalities at the peak of the outbreak, as estimated from Eqs (3) and (4). This corresponds to France, Spain, Italy, New York and New Jersey. In orders of magnitude the Google social distancing index dropped by about 80% for the European countries shown, and by about 60% for six states within the US. In Fig 7f the GSDI is shown as a function of per capita fatality rates. The general trend is that the GSDI acquires somewhat lower values for European countries, with respect to US states, together with a comparative pronounced recovery.

There is a certain spread in the total fatalities *F* needed for social distancing to be fully developed, as shown in Fig 7. In per capita terms, the GSDI dropped however fast in all countries and US state examined.

## 3 Methods

An interactive web-tool for the study of COVID-19 case and death data, the “Goethe Interactive COVID-19 Analyzer”, is available [29]. It allows in particular for country-specific phase space representations, as used widely throughout the present study. Databases of government responses to the COVID-19 outbreak, like the “Oxford COVID-19 Government Response Tracker” [30, 31], can be used to correlate specific containment policies with the evolution of the epidemic [32]. This is not done here, as we concentrate on overall attributes of containment policies, namely on short- vs. long-term control, and not on individual measures. We will maintain the interactive web-tool until the end of November 2023 (minimal duration).

### 3.1 Data sources

COVID-19 data sources used are the public GitHub repository of the Johns Hopkins Center for Systems Science and Engineering (JHU-CSSE) [33], the European Center for Disease Control open COVID-19 data (ECDC) [34], and the German Robert Koch Institute [35] (RKI). If not otherwise stated we used ECDC for country-specific data and JHU-CSSE for US states. A comparison is presented in Fig 2. Both data sets were last updated on December 8th, 2020.

### 3.2 Data smoothing / peak definition

The real-world epidemics reports are intrinsically noisy, with common sources of noise being report delays and under- or over-counting [36]. All data sources used show strong fluctuations within a seven day period. Accordingly, we utilize a seven-day centred moving average for data preprocessing.

Due to a multitude of errata in the data sets, it is also necessary to filter out impossible measurements, such as negative daily new cases or fatalities. As a remedy we dropped dates with negative daily fatalities Δ*F*_*d*_ < 0. In these isolated cases the seven-day centered moving average is evaluated over the remaining seven data points, spanning eight actual days.

In most countries the initial COVID-19 outbreak has been followed by endemic, low-level phases and second waves. The underlying reason is the return to short-term control, reaction-type policies, when the first wave has been contained to a certain extent. The here developed framework, the controlled SIR model, is based in contrast on the assumption that containment policies, in terms of *α*_*X*_ and *α*_*I*_, are constant, with the consequence that the controlled SIR model describes a single contained outbreak, and not a series of waves. However, only a well defined peak is needed for a reliable analysis. The epidemic need not be fully eradicated for a reliable analysis. Our framework is therefore well suited to analyze the first wave of a COVID-19 epidemic, during which the containment feedback parameters *α*_*X*_ and *α*_*I*_ can be assumed to not have changed substantially.

To be specific, we define a cut-off date *d*′ as the day on which the number of daily new fatalities has fallen by 70% compared to the first peak: Δ*F*_d′_ = 0.3Δ*F*_peak_. Here Δ*F*_peak_ denotes the maximum number of daily new fatalities in the 7-day centered moving average. This criterion is used to isolate the first outbreak, *d* < *d*′, from the subsequent course of the epidemic. The dates *d*′ used are listed in Table 1.

### 3.3 Google Social Distancing Index (GSDI)

The Google COVID-19 mobility data describes changes in a range of mobility-related activities, each measured with respect to corresponding Google search queries [37]. We define a “Google social distancing index” (GSDI) as the average of the three categories “workplaces”, “retail and recreation” and “transit stations”, which are given respectively by the percentage-wise activity drop relative to their pre-COVID-19 baselines. The GSDI is presented in Fig 7. In other studies, Google and smartphone mobility data has been used to correlate containment policies with social distancing [38], to identify the importance of transport nodes [39], and to quantify the impact of social status on social distancing [40].

### 3.4 Exact solution of the controlled SIR model

The phase space trajectory of the controlled SIR model, Eqs (1) and (2), can be derived expressively. For the derivation we extend an approach used elsewhere for the case of pure long-term control [22], starting with

$$\begin{array}{c} \frac{dI}{dS}=\frac{N+\alpha_{X}X+\alpha_{I}I}{g_{0}\cdot S}-1,\;\;\frac{dI}{dS}-\frac{\alpha_{I}I}{g_{0}S}=N\frac{1+\alpha_{X}}{g_{0}S}-1-\frac{\alpha_{X}}{g_{0}}\;. \end{array}$$ (10)

In order to obtain total differentials, one multiplies Eq (10) with the auxiliary function

$$\begin{array}{c} F\left(S\right)=S^{-\alpha_{I}/g_{0}},\;\;\frac{dF}{dS}=\frac{-\alpha_{I}}{g_{0}}S^{-(\alpha_{I}/g0)-1}\;, \end{array}$$ (11)

with the result

$$\begin{array}{c} F\frac{dI}{dS}+I\frac{dF}{dS}=-N\frac{1+\alpha_{X}}{\alpha_{I}}\frac{dF}{dS}-\frac{g_{0}+\alpha_{X}}{g_{0}}F\;, \end{array}$$ (12)

where the left-hand side is now equivalent to *d*(*FI*)/*dS*. Integration yields

$$\begin{array}{c} FI=-N\frac{1+\alpha_{X}}{\alpha_{I}}F\;-\;\frac{g_{0}+\alpha_{X}}{g_{0}}\frac{1}{1-\alpha_{I}/g_{0}}S^{1-\alpha_{I}/g_{0}}\;+\;C\;, \end{array}$$ (13)

or

$$\begin{array}{c} I=-N\frac{1+\alpha_{X}}{\alpha_{I}}\;-\;\frac{g_{0}+\alpha_{X}}{g_{0}-\alpha_{I}}S\;+\;CS^{\alpha_{I}/g_{0}}\;. \end{array}$$ (14)

The starting condition *I*(*S* = *N*) = 0 determines the integration constant as

$$\begin{array}{c} C=N^{1-\alpha_{I}/g_{0}}[\frac{1+\alpha_{x}}{\alpha_{I}}+\frac{g_{0}+\alpha_{X}}{g_{0}-\alpha_{I}}], \end{array}$$ (15)

which leads to the final expression

$$\begin{array}{c} I=\frac{g_{0}+\alpha_{X}}{g_{0}-\alpha_{I}}X+N(\frac{g_{0}+\alpha_{X}}{g_{0}-\alpha_{I}}+\frac{1+\alpha_{X}}{\alpha_{I}})\left[(1-\frac{X}{N}{)}^{\alpha_{I}/g_{0}}-1\right]\equiv f\left(X,\theta \right)\;, \end{array}$$ (16)

where *θ* = (*α*_*X*_, *α*_*I*_, *g*_0_). The two formal divergences on the right-hand side, *α*_*I*_ → 0 and *α*_*I*_ → *g*_0_, are well behaved. The first limit, *α*_*I*_ → 0, is obtained using

$$\begin{array}{c} (1-\frac{X}{N}{)}^{\alpha_{I}/g_{0}}={\text{e}}^{\alpha_{I}\;\text{log}(1-\frac{X}{N})/g_{0}}\approx 1+\alpha_{I}\;\text{ln}\left(1-\frac{X}{N}\right)/g_{0}\;, \end{array}$$ (17)

which reduces Eq (16) to the XI representation with long-term control [22]

$$\begin{array}{c} I{|}_{\alpha_{I}\to 0}=\frac{\alpha_{X}+g_{0}}{g_{0}}\;X+N\frac{1+\alpha_{X}}{g_{0}}\;\text{ln}\left(1-\frac{X}{N}\right). \end{array}$$ (18)

The formal divergence in the XI representation of mixed control Eq (16) occurring when *α*_*I*_ ≈ *g*_0_ cancels equivalently. To see this consider the expansion of

$$\begin{array}{ccc} (1-\frac{X}{N}{)}^{\alpha_{I}/g_{0}} & = & (1-\frac{X}{N}{)}^{\alpha_{I}/g_{0}-1+1}=\left(1-\frac{X}{N}\right){\text{e}}^{\frac{\alpha_{I}-g_{0}}{g_{0}}\text{ln}(1-\frac{X}{N})}\\ & \approx & (1-\frac{X}{N})[1+\frac{\alpha_{I}-g_{0}}{g_{0}}\text{ln}\left(1-\frac{X}{N}\right)]\;, \end{array}$$ (19)

which leads to

$$\begin{array}{c} I{|}_{\alpha_{I}\to g_{0}}\approx -\frac{1+\alpha_{X}}{\alpha_{I}}X-N\cdot \frac{g_{0}+\alpha_{X}}{g_{0}}\left(1-\frac{X}{N}\right)\text{ln}\left(1-\frac{X}{N}\right)+O(|\alpha_{I}-g_{0}{|}^{1})\;. \end{array}$$ (20)

An important point is that the starting slope of Eq (16),

$$\begin{array}{c} \frac{dI}{dX}{|}_{X\to 0}=\frac{g_{0}-1}{g_{0}}\;, \end{array}$$ (21)

is independent of both *α*_*I*_ and *α*_*X*_. For the derivation of Eq (21) one uses $(1-\frac{X}{N}{)}^{\alpha_{I}/g_{0}}\approx 1-\frac{\alpha_{I}}{g_{0}N}X$. This relation has been used to calculate *g*_0_ in Fig 7.

### 3.5 Approximate integration

Most COVID-19 datasets, contain, among other measures, the total known number of infected people *X*_*d*_ and the total number of fatalities *F*_*d*_ up to day *d*. For a period Δ*t* of one day, daily cases correspond to the change in total cases Δ*X*_*d*_ = *X*_*d*+Δ*t*_ − *X*_*d*_. Equivalently, daily fatalities are equal to the change in the total number of fatalities Δ*F*_*d*_ = *F*_*d*+Δ*t*_ − *F*_*d*_. We will consider the reports of daily fatalities more accurate in general than daily cases (or daily recovered) as under or over counting is less severe. In what follows we will demonstrate how one can relate daily cases and daily fatalities to the infection rate *g*(*t*), and to the XI representation.

Eq (1) can be expressed as

$$\begin{array}{c} \tau \dot{X}=g\frac{N-X}{N}I,\;\tau \frac{\text{d}}{\text{d}t}\text{ln}\;I=\left(g\frac{N-X}{N}-1\right),\;\tau \dot{R}=I\;. \end{array}$$ (22)

Integrating Eq (22) between *d* and *d* + Δ*t* we obtain

$$\begin{array}{c} \begin{array}{cc} \Delta X_{d} & =\frac{1}{\tau }\int_{d}^{d+\Delta t}g\left(t^{\prime }\right)(\frac{N-X(t^{\prime })}{N})I\left(t^{\prime }\right)\text{d}t^{\prime }\\ I_{d+\Delta t} & =I_{d}\text{exp}[\frac{1}{\tau }\int_{d}^{d+\Delta t}\left(g\left(t^{\prime }\right)\frac{N-X(t^{\prime })}{N}-1\right)\text{d}t^{\prime }]\\ \Delta R_{d} & =\frac{1}{\tau }\int_{d}^{d+\Delta t}I\left(t^{\prime }\right)\text{d}t^{\prime } \end{array} \end{array}$$ (23)

Following the approximate integration steps presented in [41], we assume that the quantities of interest, *X*(*t*), *I*(*t*), and *g*(*t*) are piecewise constant within [*d*, *d* + Δ*t*). Hence, after setting the integration interval to one day, Δ*t* = 1, we get the following set of difference equations

$$\begin{array}{c} \begin{array}{cc} I_{d+1} & \approx I_{d}\text{exp}[\frac{1}{\tau }(g_{d}\frac{N-X_{d}}{N}-1)]\\ \Delta X_{d} & \approx \frac{1}{\tau }g_{d}\left(N-X_{d}\right)I_{d}\\ \Delta R_{d} & \approx \frac{1}{\tau }I_{d} \end{array} \end{array}$$ (24)

Individuals are removed, *ΔR*_*d*_, after recovery or death, which implies Δ*F*_*d*_ = *c*_*F*_Δ*R*_*d*_, or when quarantined, Δ*Q*_*d*_ = *c*_*Q*_Δ*R*_*d*_. We denote here with *Q*_*d*_ the number individuals that are infected, but unable to infect others, either because they are quarantined at home, or because they are hospitalized. In general *c*_*F*_ + *c*_*Q*_ < 1 and *c*_*Q*_ > *c*_*F*_. Using Eq (24) we obtain two approximate relations for the evolution of daily quarantined and deaths,

$$\begin{array}{c} \begin{array}{c} \Delta Q_{d}\approx \left(c_{Q}/\tau \right)I_{d}\\ \Delta F_{d}\approx \left(c_{F}/\tau \right)I_{d} \end{array}\;. \end{array}$$ (25)

In practice, people tested positive will be advised to quarantine, or hospitalized. In view that the officially reported new cases, Δ*X*_*d*_, correspond to the number of positive COVID-19 tests outcomes, one has, with Eq (25), that Δ*X*_*d*_ ∼ Δ*Q*_*d*_ ∼ *I*_*d*_ ∼ Δ*F*_*d*_, and hence that Δ*X*_*d*_ scales approximately with Δ*F*_*d*_. We believe that this reasoning explains the observed approximate scaling between case- and death counts, as shown in Fig 3.

**Fig 8:**
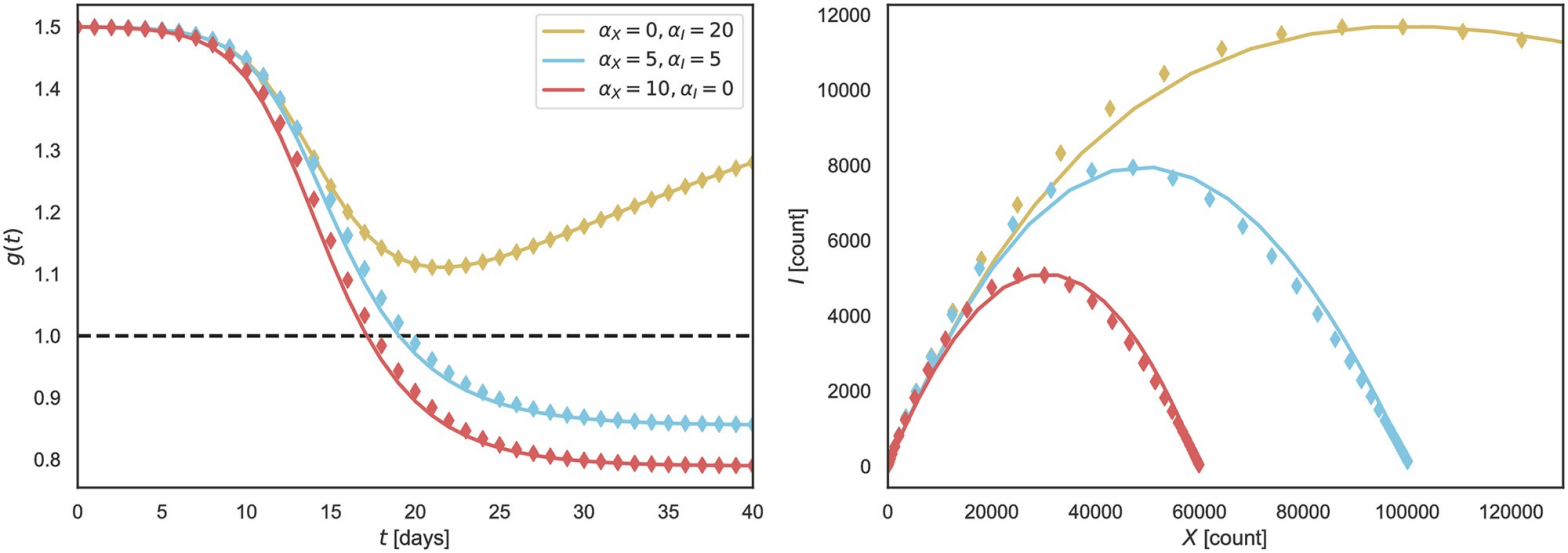
Solutions of the controlled SIR model vs. simulated data. Comparison of *g*(*t*) and *I*(*X*) obtained numerically (solid lines) and from simulated daily fatalities (diamonds). See Eq (2) for the effective reproduction factor *g* = *g*(*t*) and Eq (16) for the XI representation. Simulated daily and total fatalities were obtained from active cases as Δ*F*_*d*_ ∝ *I*(*t* = *d*), and $F_{d}=\sum_{d^{\prime }=1}^{d}\Delta F_{d}$. Hence diamonds correspond to a points for which $X_{d}=\sum_{d^{\prime }=1}^{d}I_{d^{\prime }}$. Similarly, the recovered *g*_*d*_ are obtained from the following relation $g_{d}=(\tau \;\text{ln}\frac{I_{d+1}}{I_{d}}+1)\cdot (\frac{N-X_{d}}{N})$. Identical initial conditions have been used for all cases, *X*(0) = 10, *I*(0) = 10, *R*(0) = 0, *g*(0) = 1.5, and *N* = 10^6^. See Approximate integration for details.

As a further test of the procedure outlined above we compare in Fig 8 the solution of the controlled SIR model with simulated data. Here we obtained *g* = *g*(*t*) by numerical integrating Eq (1), with the phase space representation matching the analytic expression, Eq (3). Using Eq (25) the timeline of infected, *I*(*t*), was used to generate simulated data for daily fatalities, Δ*F*_*d*_, which in turn yields the cumulative death count *F*_*d*_ = ∑_*d*″≤*d*_ Δ*F*_*d*″_. As a last step we rescaled via Eq (7), $\left(X,I\right)≙{\tilde{f}}_{F}\left(F,\Delta F\right)$, comparing the simulated data, (*X*, *I*), with the direct solution of the controlled SIR model. The agreement between the direct solution and simulated data is remarkably good.

### 3.6 Parameter estimation

To fit the parameters of the controlled SIR model to the publicly available outbreak datasets, we have used the theoretical phase relation Eq (3). The best fitting parameter values are obtained by direct minimization of the following loss function

$$\begin{array}{c} U=\sum_{d}w_{d}[f_{F}\Delta F_{d}-I(f_{F}F_{d}){]}^{2}\;, \end{array}$$ (26)

where the weights *w*_*d*_ = *F*_*d*_ − *F*_*d*−1_ = Δ*F*_*d*_ ensure that long stretches of days with low fatality numbers, which are common at the beginning and the end of an epidemic, do not dominate. An optimization that weighs every data point equally, i.e. least squares, overestimates the early and in the case of one isolated outbreak the late stages of the epidemic outbreak. The rescaling factors *f*_*F*_ used are the ones presented in Table 1.

The minimization of Eq (26) with respect to the parameter set {*α*_*X*_, *α*_*I*_, *g*_0_} has been performed using Newton‘s method for optimization implemented in Julia by [42]. To prevent division by zero the denominators of Eq (3) have been shifted by *εi* = 0.01*i* in the complex plane before taking the real part.

### 3.7 Simulation

The theoretical values of the ratio of average cases after and before the infection peak presented in Fig 6 were calculated simulating the controlled SIR model Eq (1). The numerical integration was performed using the SciMl implementation of Jim Verner’s “most efficient” 7/6 Runge-Kutta method [43]. Unless stated otherwise, the simulations were run for *τ* = 1.1 and *g*_0_ = 1.25. The population has been initialized with *I*(*t* = 0) = 10^−10^ *N*. For every *α*_*X*_ and *α*_*I*_ we calculated the peak medical load *I*_peak_ using Eqs (3) and (4) exact. The percentage-wise contribution *L*/(*L* + *S*) of long-term control Eq (8) are evaluated at the point of peak infection rates.

Note that it is not possible to simulate different trajectories for which both *L*/(*L* + *S*) and *I*_peak_/N are fixed. For the comparison presented in Fig 6 simulations with varying *α*_*X*_ and *α*_*I*_ were used to bin the resulting *L*/(*L* + *S*) and *I*_peak_/N within about 0.5% accuracy.

## 4 Discussion

By mid 2020, the world-wide COVID-19 pandemic has entered a phase, where the initial exponential growth phase has been contained in most countries and regions to the extent, that official case counts dropped substantially with respect to the first peak. For the majority of countries and regions it is therefore possible to define an endpoint *d*′ of the first wave. Here we used a simple criterion, namely a 70% drop in case numbers. Subsequent to the first wave, the development of the SARS-CoV-2 pandemic is showing a large variety of functional dependencies.

For a large number of COVID-19 outbreaks we analyzed the first wave in terms of an effective SIR model, the controlled SIR model. The basic assumption is that containment policies can be parametrized by two parameters, *α*_*X*_ and *α*_*I*_, which describe how much emphasis is placed respectively on long- and short-term control. This does not imply that containment in terms of a reduction of the basic reproduction factor is constant, but that the dependence of the reproduction factor on total and daily case counts is given by a functionally constant feedback loop. For a wide range of countries and US states we find that the official case and death counts are described well by the controlled SIR model. This observation allows us to extract country-specific containment parameters, *α*_*X*_ and *α*_*I*_. Containment success is found to go hand in hand with an emphasis on long-term control, with short-term control being more likely to be followed by an endemic state.

Two types of time lines can be used to analyse COVID-19 outbreaks, one based on daily cases and the other based on daily fatalities. In this study we examined in particular the death toll, showing that daily and cumulative fatalities provide reliable data sources. This framework is based on the assumption that the success of medical therapies does not change substantially over the course of the observation period, here the first wave. Given the accuracy of the modeling, this assumption sees a posteriori justification, which is further strengthened by the observation that case and fatality counts scale in phase space representation, as shown in Fig 3.

A particularly interesting result of our analysis concerns the predictability of the medical load. As a measure we compare the cumulative number of fatalities over two periods of identical length, typically several weeks, just before and just after the peak of the first wave. In this regard, we find that the medical load increases on average by 30% after the peak. This is quite a remarkable observation, in our view, given that the COVID-19 outbreaks vary substantially in between countries.

Detailed epidemiological modeling is necessary in particular when examining specific scenarios, like the effect of school opening strategies [44]. Given that it is often difficult to estimate the respective parameters reliably [1, 2], we opted here for an approach based on effective modeling theory. This framework allows to examine the statistics of COVID-19 deaths directly in a phase space representation, as done in Fig 4. The alternative, full epidemiological modeling, would need to go through the statistics of a larger number of compartments describing exposed individuals, symptomatic and asymptomatic infections, quarantined, etc. In some cases, e.g. for the daily number of asymptomatic infections, there are no publicly available reliable databases. This is not a problem for purely theoretical studies that examine the consequences of certain parameter constellations [45]. Although it is possible to determine additional quantities indirectly, like the percentage of asymptomatic cases, by fitting to official case counts [46, 47], this would introduce in general increased uncertainties to the estimates of relevant model parameters, making the entire procedure strongly susceptible to the quality of the underlying data.

Here we argue that certain database problems, like reporting delays and under-counting, can be circumvented by focusing on daily fatalities and using the phase space (XI) representation of a the controlled SIR model. Still, the presented analysis is not without limitations. For example, we did not take into account the possibility of regime changes, that is, parameter changes over time. The long-term and short-term reaction parameters could in practice evolve, as society adapts to the new information about the outbreak. Similarly, reporting delays for daily cases and fatalities might get reduced with time as the governmental administration improves the reporting process. Importantly, incorporation of assumptions about the data generating process to the data analysis method, will definitely bring more precision and robustness to model fitting process and the parameter recovery. We leave such extended analysis for future work, as the availability of more data about the pandemic in the future would also allow considering more complex models.

## Supporting information

S1 Fig Comparison of long-term control components. As in Fig 4, the two countries/regions with highest (Turkey) and lowest (USA/Georgia) fraction *L*/(*L* + *S*) of long-term control. Compare Table 1.
(TIF)

S1 File Processed data for daily fatalities, daily cases, and Google Social Distancing Index (GSDI). (ZIP)
