# Supplementary figures and images for: Predicting the cumulative medical load of COVID-19 outbreaks after the peak in daily fatalities
Source: PLoS One. 2021 Apr 1;16(4):e0247272. doi: 10.1371/journal.pone.0247272 (PMC8016333; doi:10.1371/journal.pone.0247272)

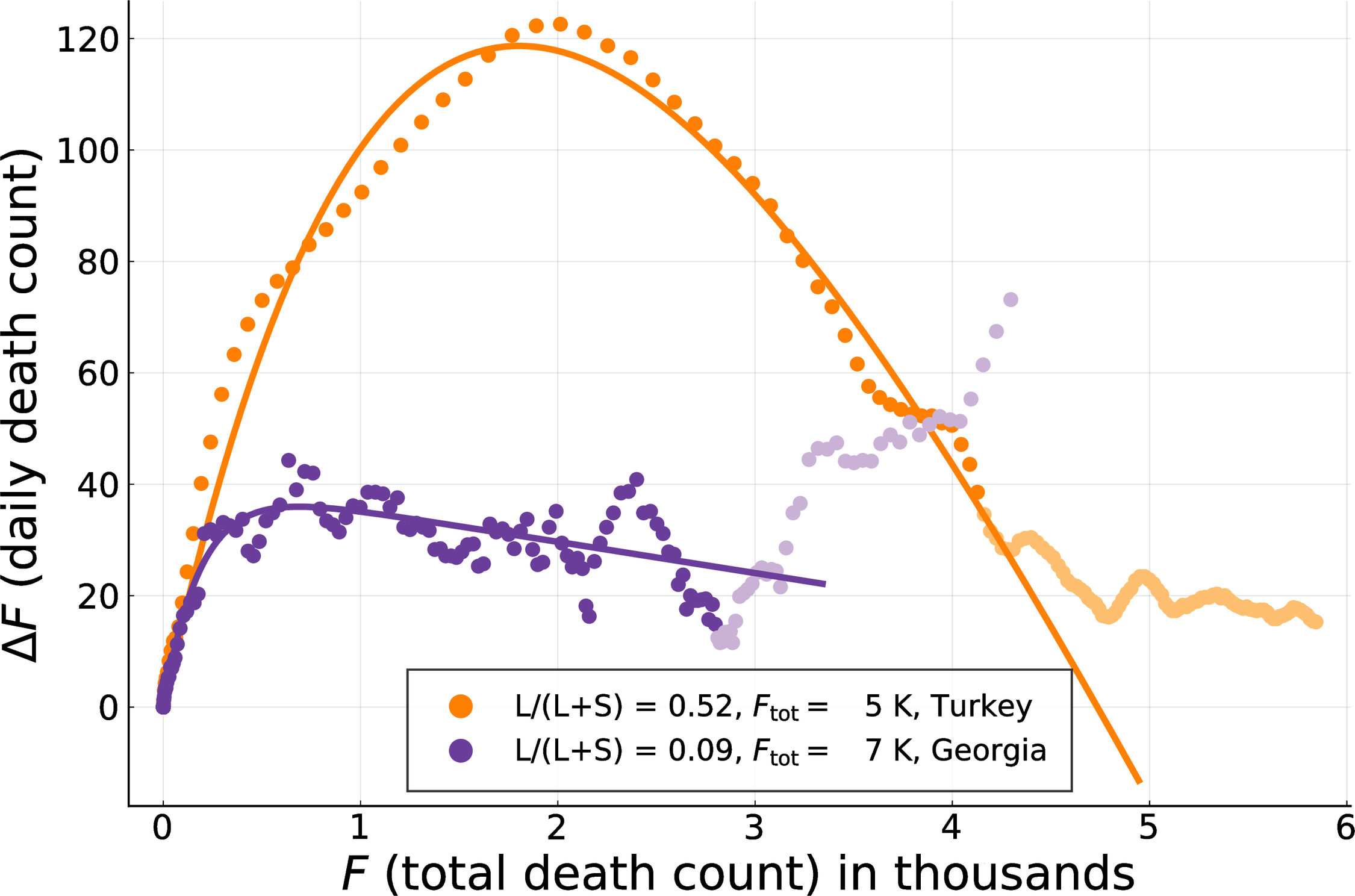

Supplement: S1 Fig — As in Fig 4, the two countries/regions with highest (Turkey) and lowest (USA/Georgia) fraction L/(L + S) of long-term control. Compare Table 1. (TIF) [file pone.0247272.s001.tif]
